# Perceptions of effective leadership in a medical school context

**Published:** 2019-07-24

**Authors:** Aleem Bharwani, Theresa Kline, Margaret Patterson

**Affiliations:** 1 Cumming School of Medicine, University of Calgary, Alberta, Canada; 2 Department of Psychology, University of Calgary, Alberta, Canada; 3 Werklund School of Education, University of Calgary, Alberta, Canada

## Abstract

**Background:**

There have been calls for the development of leadership attributes in healthcare practitioners through leadership development programs. However, understanding how leadership is conceptualized is needed to assure effective participant-centred leadership development programs. The purpose of this study was to elucidate how the construct of leadership is conceptualized by multiple stakeholder groups associated with medical school leadership programs.

**Methods:**

We conducted a total of 77 semi-structured interviews with six major demographic groups: Trainees (*n* = 16), Mid-Level University Leaders (*n* = 10), Clinician Leaders (*n* = 17), Senior University Leaders (*n* = 10), Medical Scientists (*n* = 12), and Senior Leaders, external to the University (n = 12) to address the research question.

**Results:**

Content analyses revealed that the leaders were expected to create a compelling vision and a foster a motivating culture within the organization. Integrity and a sense of passion about leading were viewed as being principal characteristics of a leader. The twin skills of technical competence and communication were endorsed as most important for a leader. Finally, leaders are expected to be accountable for outcomes.

**Conclusion:**

Medical school leadership training programs should strive to incorporate these characteristics, given their broad appeal to diverse interest groups.

## Introduction

There is a clear need to ensure that leadership is functioning in healthcare settings^1^ and medical school training programs have endeavored to develop such competencies.^2^ In this study we strove to understand how the construct of leadership is conceptualized by the various stakeholder groups (learners, teachers, sponsors) of such programs, as the need for participant-centered leadership development programming is critical to its success.^3^

Training programs are more successful if there is end- user acceptance of the underlying rationale of the program by the participants.^4^ People’s perceptions of what makes an effective leader will influence their expectations about the content of a leadership development program.^5^ If trainees’ expectations about the program are not met, they are less likely to have positive attitudes toward the program.^6^ Thus, it is important to understand what different stakeholder groups’ perceptions are of an effective leader.^7^ We used a qualitative approach to determine how effective leadership was defined by six different stakeholder groups in a medical school setting.

## Methods

### Sample

Since we included six different stakeholder groups, we created an initial sample of 69 individuals from among these groups, based on their interest in leadership, as personally expressed to the principal investigator. We sent email invitations to each of these individuals and followed up with those who accepted the invitation to schedule their interviews. Interviewees were primarily from medical schools and universities in Alberta and British Columbia, directors of health agencies or other organizations with ties to the University of Calgary, and one was a CEO from the United States interested in leadership.

We used snowball sampling to secure subsequent potential participants, requesting interviewees to indicate others they knew with an interest in leadership in a health care context. We sent out 161 email invitations and 77 people agreed to participate in the study (response rate of 48%). We reached theme saturation after about seven interviews per group, but conducted a few additional interviews to be sure.

We sampled six major demographic groups:

- Trainees (medical students, graduate students, residents, fellows, post-doctoral researchers) (n = 16)
- Mid-Level University Leaders (associate deans, assistant deans, institute directors) (n = 10)
- Clinician Leaders (physicians who were also medical directors or department heads) (n = 17)
- Senior University Leaders (provosts, deputy provosts, deans, vice deans) (n = 10)
- Medical Scientists (professors, scientific directors) (n = 12)
- Senior Leaders, external to the University (presidents, vice-presidents, boards of directors) (n = 12)

Eight different interviewers, (faculty members, research assistants and graduate students) conducted 77 semi-structured interviews (between 4- 19 each). One of the investigators trained all interviewers in interviewing techniques. We sent participants the interview questions in advance for consideration. Interviewers transcribed their own interviews.

The Conjoint Heath Research Ethics Board, University of Calgary approved the study (ID# 13-0308) and we obtained written, informed consent from all participants.

### Interview Question

This study focused on one question from a longer interview regarding the development of a leadership program: “What do you think makes an effective leader?” The responses to other questions from the longer interview addressed strategies to support leadership development^8^ and the contextual variables facilitating or hindering leadership program implementation.^9^ We conducted the interviews face- to-face (n = 61), by telephone (n = 15) or by Skype (n = 1) between December 2013 and May 2014; they lasted an average of 34 minutes (range 11 to 140; SD 19).

### Analysis

We analyzed the interview data using conventional content analysis.^10^ Words, simple sentences, or strings of words expressing a single thought formed a theme and were the unit of analysis. We constantly compared and reviewed/revised the themes after introducing new text, either adding the new text as a further exemplar of an existing theme or creating a new theme. We formed higher-level categories based on themes with common underlying characteristics. Categories had to be both comprehensive and mutually exclusive.

One investigator trained two research assistants (not the interviewers) in the coding protocol. One of the research assistants coded the data and the second verified the coding. They discussed any discrepancies until they reached unanimous agreement. We then reviewed all coded themes and categories; we unanimously agreed on any further changes made to the coding.

## Results

To keep within the scope of a brief report, we report on themes that emerged from at least 33% of at least one stakeholder group. Comments from all stakeholder groups related to four general categories: Leadership Actions; Traits and Characteristics; Knowledge and Skills; and Focus on Outcomes. See Table 1 for further details.

**Table 1:** Categories and themes

Theme	Trainees	Mid-Level University Leaders	Clinical Leaders	Senior University Leaders	Medical Scientists	Senior External Leaders	Overall
	16*	10	17	10	12	12	77
**Category: Leadership Actions**							
Create a Compelling Vision	.56†	.70	.47	.60	1.00	.58	.64
Create a Motivating Culture	.69	.40	.47	.60	.83	.50	.58
Work Hard	.63					.50	.21
Recognize and Reward/Talent Management				.30	1.00		.19
Build Consensus	.44			.30			.13
Engage Employees		.40	.12	.14			.10
Clear Decision-Making Process		.70					.09
Be Politically Savvy		.70					.09
Be Proactive					.33		.05
**Category 2: Traits/Characteristics**							
Honest/ Integrity/ Ethical/ Trustworthy	.19	.40	.41	.40	.90	.17	.40
Passionate/ Inspiring/ Energetic	.31	.40	.41	.30	.67	.25	.39
Respectful/ Humble	.50	.20		.60	.50		.29
Emotionally Intelligent		.30	.24		.83	.33	.27
Authentic/ Self-Awareness	.25		.41	.20		.42	.23
Engaging	.63	.30	.29				.23
Open	.38		.35		.25		.20
Agreeable	.38					67	.18
Selfless/ Altruistic		.06			.33	.25	.10
Compassionate	.13	.06			.33		.09
Conscientious	.44						.09
Problem Identifier						.58	.09
Willing to Lead/ Committed		.40		.20			.08
Reflective		.10		.40			.06
**Category 3: Skills/Knowledge**							
Communication Skills	.69	.50	.59	.60	.67	.75	.64
Technical Competence	.31	.30	.41	.30	.50	.50	.39
Building Relationships/ Diplomacy/ Collaborate	.94	.10			.25		.25
Manage Teams		.10	.35	.30	.67		.23
Personal/Time Management/ Organizational Skills	.38				.58	.08	.18
Decision-Making Skills			.41		.17		.12
Strategic Thinking				.40	.58		.14
Organizational Knowledge	.18		.41				.13
Selecting a Supportive Team	.56						.12
Stay Connected to the Front Line	.44						.09
Understand Motivations						.42	.07
**Category 4: Focus on Outcomes**							
Accountable for Outcomes	.06	.10	.23	.10	.08	.33	.15

* Sample sizes are the number of participants in each stakeholder group.

† Cell values indicate the proportion of participants from each stakeholder group who made comments that fit the theme.

### Leadership Actions

Our participants expected leaders to provide a compelling vision of the future and be able to communicate that to their followers. An exemplary comment on this action is: “*The way in which the vision is presented is critical. It has to be presented in a … way so the group can see themselves as part of it*.” Participants also expected leaders to be at the forefront of creating a culture that supports leadership development. A comment on doing do is: *“Encourages others to develop leadership skills and take on leadership positions.”*

After these first two themes, there was some divergence in the actions expected of effective leaders depending on the stakeholder group. Both Trainees and Senior External Leaders mentioned working hard as an important action. Senior University Leaders, and especially Medical Scientists, demanded leaders recognize and reward talent appropriately. Trainees and Senior University Leaders noted that building consensus was an important behaviour. Mid-Level, Clinical, and Senior University Leaders expected employee engagement, suggesting that at the upper levels, there is an expectation of involvement in the leadership process. Mid-Level University Leaders also expected leaders to have a clear decision-making process and be politically astute, reflecting perhaps their position in the organizational hierarchy. Medical Scientists expected to see proactive behaviours by leaders.

### Leadership traits and characteristics

All of the stakeholder groups mentioned two leadership traits and characteristics themes. The first was honesty and integrity and the other was the ability to inspire. Four different stakeholder groups noted the importance of these traits: respectful/humble, emotionally intelligent, and authentic/self-aware.

### Skills and knowledge

The third characteristic associated with an effective leader was their skills and knowledge. All stakeholder groups spoke strongly about the need for leaders to have both communication skills and technical competence. Other skills that emerged as important were: building relationships, managing teams, and time/organizational management skills. Divergence occurred at this point in skills deemed most relevant including: decision-making skills, strategic thinking, and knowledge about the organization.

### Focus on outcomes

The fourth category mentioned by all stakeholder groups was that leaders need to be accountable for organizational outcomes.

## Discussion

Our findings are similar to those in a study of cross- cultural perspectives of leadership.^5^ This convergence about what makes an effective leader can help build the foundation of a broadly acceptable medical school leadership program.

We found, as has been noted by others, that leaders must be at the forefront of culture change^11^ requiring the articulation and communication of a compelling vision. However, leaders need be proactive in managing aspects of possible resistance to change by planning for it, providing support and training, and communicating the rationale and expected outcomes of such change.^12^ The Canadian Medical Association concurs, calling for physician leaders “to envision their preferred future for the profession and lead others toward this vision” (p. 69).^13^

The importance of honesty and integrity was underscored by our stakeholders and is consistent with literature that points out the pivotal role of trust in the leadership process.^14^ However “radical honesty” – transparent and candid - can be uncomfortable. This may be particularly true in performance evaluations where skilled delivery of feedback is needed.^15^ Respondents also consistently noted that being able to inspire others was important. This is a cross-cultural aspect of leadership.^5^ Selecting individuals with the traits of honesty and integrity as well as an inspiring communication style to participate in a leadership program would likely enhance their chances of being successful, and thus support the program’s success.

Our participants asserted that a leader is expected to be clinically competent to assure credibility.^16^ However excellent clinical skill, while important is not enough on its own to ensure effective leadership.^17^ Developing good communication skills is a must,^18^ and therefore has to be a focus of competency development of any leadership program.

The fourth category emphasized the importance of accountability for outcomes. This is consistent with literature that stresses leadership effectiveness is judged by outcomes.^19,20^

The facets of behaviours, personal characteristics, skills, and the ability to motivate others to accomplish organizational goals that make up a good leader do not necessarily act independently of one another. A program that integrates the development all these competencies in their leaders enhances the capacity for synergy between them.^21^

### Conclusion

Leadership training program success depends on offering the right development to the right people.^22^

Our respondents held some similar perceptions regarding what makes as effective leader including their actions, personality traits, and skills sets. A leadership development program at medical schools should include: 1) selection of candidates based on leadership traits that can be nurtured; 2) development of: a) the capacity for creating a compelling vision, b) technical skills and, c) communication skills and 3) accountability for meeting goals. Such a program would have broad appeal among most stakeholder groups.
